# Bacteria Associated with Acute Oak Decline: Where Did They Come From? We Know Where They Go

**DOI:** 10.3390/microorganisms11112789

**Published:** 2023-11-17

**Authors:** Daniel Maddock, Carrie Brady, Sandra Denman, Dawn Arnold

**Affiliations:** 1 Centre for Research in Bioscience, College of Health, Science and Society, University of the West of England, Bristol BS16 1QY, UK; daniel.maddock@uwe.ac.uk; 2 Centre for Ecosystems, Society and Biosecurity, Forest Research, Farnham GU10 4LH, UK; sandra.denman@forestresearch.gov.uk; 3 Harper Adams University, Newport TF10 8NB, UK; darnold@harper-adams.ac.uk

**Keywords:** acute oak decline, AOD, niches, enrichment, recovery, acorns, leaves, rhizosphere

## Abstract

Acute oak decline is a high-impact disease causing necrotic lesions on the trunk, crown thinning and the eventual death of oak. Four bacterial species are associated with the lesions—*Brenneria goodwinii*, *Gibbsiella quercinecans*, *Rahnella victoriana* and *Lonsdalea Britannica*—although an epi-/endophytic lifestyle has also been suggested for these bacteria. However, little is known about their environmental reservoirs or their pathway to endophytic colonisation. This work aimed to investigate the ability of the four AOD-associated bacterial species to survive for prolonged periods within rhizosphere soil, leaves and acorns in vitro, and to design an appropriate method for their recovery. This method was trialled on field samples related to healthy and symptomatic oaks. The in vitro study showed that the majority of these species could survive for at least six weeks within each sample type. Results from the field samples demonstrated that *R. victoriana* and *G. quercinecans* appear environmentally widespread, indicating multiple routes of endophytic colonisation might be plausible. *B. goodwinii* and *L. britannica* were only identified from acorns from healthy and symptomatic trees, indicating they may be inherited members of the endophytic seed microbiome and, despite their ability to survive outside of the host, their environmental occurrence is limited. Future research should focus on preventative measures targeting the abiotic factors of AOD, how endophytic bacteria shift to a pathogenic cycle and the identification of resilient seed stock that is less susceptible to AOD.

## 1. Introduction

The current episode of acute oak decline (AOD) was first reported in Great Britain around 2008 on native British oaks. In more recent years, the disease has spread throughout England and has been reported in much of Europe and as far as Iran on multiple species of oak [1,2,3,4,5,6]. Due to the complex nature of decline diseases, large amounts of research have taken place to understand both the biotic and abiotic factors that contribute to the disease and the overall death of the afflicted oaks. A major outcome of this research is the updated decline disease spiral model, which highlights the predisposition of trees to disease through changes in the soil, climate, pollution, primary root pathogens, management, tree age and genetic potential. Predisposition combined with inciting factors such as repeated attacks on the foliage, disturbance and prolonged droughts or floods, followed by contributing factors, namely biotic pathogens, force the tree from a healthy state into severe decline and eventually death [7]. The biotic components involved in the oak stem lesions are a polymicrobial complex of bacteria that lead to the weeping lesions that are symptomatic of AOD [8]. The four most commonly isolated species from lesions that have fulfilled Koch’s postulates are *Brenneria goodwinii, Gibbsiella quercinecans, Rahnella victoriana* and *Lonsdalea britannica* [9]. Of these bacteria, *B. goodwinii* has been identified as the core member of the lesion microbiome through both pathogenicity trials on logs [8] and genome-database comparisons, which highlighted its repertoire of virulence genes that enable its ability to thrive on oak stem tissue [10]. Meanwhile, *G. quercinecans* and *R. victoriana* appear to be secondary members of the lesion microbiome that increase the severity of lesions through several genes but are not essential to the formation of the lesion [8,10]. *B. goodwinii, G. quercinecans* and *R. victoriana* have been reported occasionally from the stem tissue of apparently healthy oak, as well as the non-symptomatic tissues in diseased oak trees [11]. Less is known about *L. britannica* due to its infrequent isolation compared to the other three members of the lesion microbiome, although other members of the genus *Lonsdalea,* such as *L. populi*, are aggressive pathogens on woody hosts [12].

Currently, isolation and lab-based cultivation of these bacteria have mostly been associated with AOD-symptomatic oak tissue [9,11], and molecular approaches have yielded differing results, with some studies failing to identify the four key species from healthy oak tissue [13,14]. However, in a more recent study by Gathercole et al. [14], non-oak sequence reads were extracted from metagenomic sequences of the oak phyllosphere and queried against bacterial databases. Sequence reads were identified for all four AOD-associated bacteria, regardless of site or tree health status, implying the AOD lesion bacteria are widely spread as endophytic or epiphytic bacteria in oak leaves [15]. However, if these bacteria are endo/epiphytes, it is still not clear where they reside in a dormant state and how they become epi/endophytic in leaves, as reported by Gathercole et al. [15], or on stems, as reported by Denman et al. [11].

The knowledge that these bacteria are associated with oak prior to disease helps to narrow down their habitat. It is important to understand where phytopathogenic bacteria originate from, or where they reside and remain dormant prior to infecting host tissues, as this can be the first target to prevent dispersal of the bacteria and therefore the spread of disease. Some bacteria can reside and remain dormant on the surface of their hosts as epiphytes until conditions become suitable and they can infect their hosts. For example, *Erwinia amylovora* overwinters in cankers on apple and pear trees and is spread to flowers in the spring; *Xanthomonas* species are able to survive in plant debris or in the soil until infection of their host; and *Liberibacter* species are phloem-limited phytopathogens that can reside as an endophyte in plant tissue [16,17]. Endophytic bacteria tend to have three dominant routes of colonisation of plant tissue. The majority of endophytic bacteria are thought to be a subpopulation of those inhabiting the rhizosphere, that enter the plant via the roots and adapt to the internal environment through altered metabolism [18]. Others are inherited as core members of the seed microbiome and are inherently endophytic at the point of germination [19], or bacteria from the environment can enter through the young leaves of plants, where they remain close to the surface in the phyllosphere [20]. The current understanding of the foliar habitat of the AOD-associated bacteria implies that the bacteria may either be endophytic or epiphytic in nature, residing in plant tissue in low numbers. However, their reservoir before dispersal into leaves is still debated, and as such, the mechanisms and pathways of dispersal and infection are not well understood.

By studying the survival of different species in diverse environmental niches and their identification in natural samples, potential origins for the AOD bacteria can be predicted, allowing insights into their endophytic nature within oak trees. This research aimed to investigate whether the bacteria associated with AOD remain dormant and survive naturally in the rhizosphere, phyllosphere, or seed endosphere, providing a foundation for their endophytic behaviour in oak trees. The objectives were to develop a method for isolating smaller quantities of these bacteria and testing their survival both in vitro and in planta. To assess the viability of these bacteria in various environments, such as soil, leaves, and acorns, the microcosms were artificially spiked with the bacteria, and subsequent re-isolation over a six-week period allowed us to identify suitable environments and isolation methods. This method was then applied in an isolation study using samples collected from Hatchlands Park, Guildford, to identify the environmental niches within the oak trees. The findings of this study contribute to a better understanding of AOD bacteria and their behaviour in different ecological contexts.

## 2. Materials and Methods

### 2.1. Strains Utilised

The type strains of *B. goodwinii* (FRB 141^T^), *G. quercinecans* (FRB 97^T^), *R. victoriana* (FRB 225^T^) and *L. britannica* (FRB 18^T^) were used in both the spiking experiment and as reference strains to generate positive high-resolution melt (HRM) curves [21,22]. Strains were stored as 50% glycerol stocks for the duration of the experiment and routinely grown in Luria-Bertani (LB) broth and agar (Oxoid, Basingstoke, UK), Gifu Anaerobic Medium (GAM, Trafalgar Scientific, Leicester, UK) and Eosin Methyl Blue (EMB, Sigma-Aldrich, Gillingham, UK) agar at 30 °C for 48 h.

### 2.2. Surface Sterilisation of Oak Material

To ensure only endophytic bacteria were identified in leaves and acorns, surface sterilisation of the plant material was performed in both in vitro and in planta experiments. Samples were submerged in 70% ethanol for 1 min followed by 10% sodium hypochlorite for 1 min. Samples were then washed twice in sterile distilled water to remove sterilising agents before use.

### 2.3. Spiking of Microcosms with AOD Bacteria

An adapted spiking method from Pettifor et al. [23] was used where bacteria were grown overnight to an OD_600_ of 0.5 in 10 mL of LB to ensure they were in the log phase, and therefore in a sufficient state to inoculate into a new medium. Overnight broths were centrifuged at 8346× *g* to pellet bacterial growth, the supernatant was removed, and the pellet was resuspended in ¼ Ringers (Oxoid). This process was repeated to ensure all LB media were removed before final resuspension. The optical density of the pure washed culture was adjusted to an OD of 0.5 by diluting with ¼ Ringers. The Miles and Misra drop-plate method was used to identify the CFU of each bacterial species that was spiked into the microcosms.

(a) To spike soil: 500 µL of pure washed bacterial culture was pipetted into the middle of a Falcon tube containing 10 g of rhizosphere soil collected from an oak located on the University of the West of England, Frenchay Campus. The soil microcosms were then shaken on the vortex and hand-shaken to ensure the dispersal of bacteria throughout the soil.
(b) To spike leaves: hypodermic needles were used to inject a total of 20 µL of pure washed bacterial culture into the petiole and midrib of *Quercus robur* leaves at three and four different points on the leaf, respectively.
(c) To spike acorns: a hypodermic needle was used to break through the pericarp and testa of *Q. robur* acorns at four symmetrical points around the centre to inject 5 µL at each point for a total of 20 µL.

**Figure 1:**
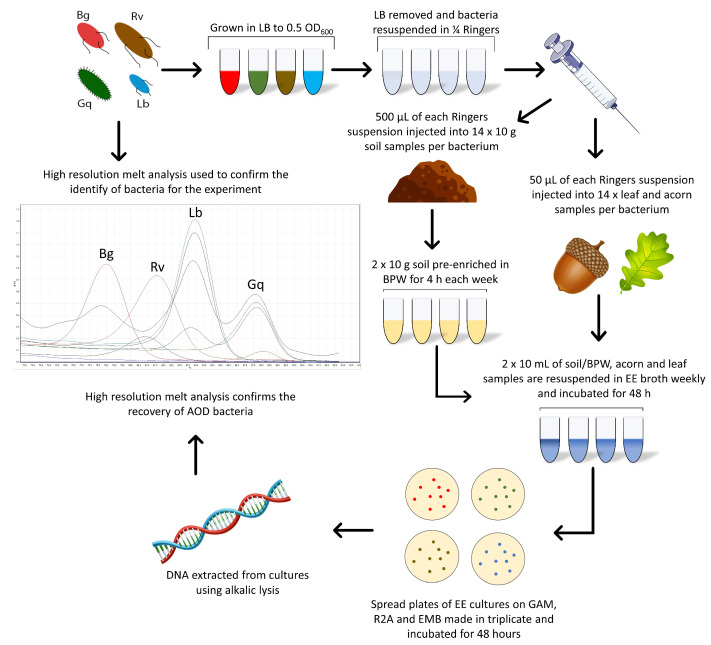
The combined methods workflow used for the spiking of bacteria into oak-related microcosms and the *Enterobacteriaceae* enrichment method used for their recovery.

Each microcosm was inoculated in duplicate for each bacterial species in a series of seven, one for each week of the experiment and the 0 h time point. Two acorn, leaf and soil samples remained uninoculated throughout the experiment to act as a control. Following the initial spiking of all microcosms, a 0 h time point measurement was taken for each sample to ensure that bacteria were not lost upon inoculation. All microcosms were stored at 8 °C, the average annual UK forest temperature [24]. One series of microcosms was removed weekly for enriched recovery of the AOD bacteria during the six-week experiment. Figure 1 details this method, leading into the enrichment method.

### 2.4. Enterobacteriaceae Enrichment of Bacteria from Microcosms

To re-isolate the spiked bacteria from the microcosms, *Enterobacteriaceae* Enrichment (EE) broth (Sigma-Aldrich) was used for the specific culturing of members of the order Enterobacterales. Ten g of soil was suspended in 95 mL of EE broth and mechanically disrupted at 1150 RPM with a Teflon-coated bar on a magnetic stirrer for 10 min. Leaf and acorn samples were ground using an autoclaved, UV-treated mortar and pestle and then suspended in the EE broth. All EE suspensions were placed in a shaking incubator for 48 h at 28 °C at 190 RPM, after which appropriate dilutions were made (10^−^⁵ and 10^−^⁶ for anaerobic and aerobic, respectively), and 100 µL was plated on EMB, LB, GAM and Reasoner’s 2 agar (R2A, Fisher Scientific, Loughborough, UK) media and incubated for 48 h at 28 °C under both aerobic and anaerobic conditions. Identification of the cultured bacteria was confirmed by a species-specific high-resolution melt (HRM) identification technique.

For *Brenneria* and *Lonsdalea*, which are more difficult to isolate in low numbers, pre-enrichment in Buffered Peptone Water (BPW) prior to re-isolation was performed. Samples were disrupted in the same manner as the EE method but were first suspended in 95 mL of BPW (Oxoid). BPW suspensions were placed in a shaking incubator for 4 h at 28 °C at 190 RPM, after which 10 mL was transferred into 90 mL of EE broth. At this point, samples in the EE broth followed the same method as described above, with samples incubated for 48 h (Figure 1).

### 2.5. Sample Collection

**Figure 2:**
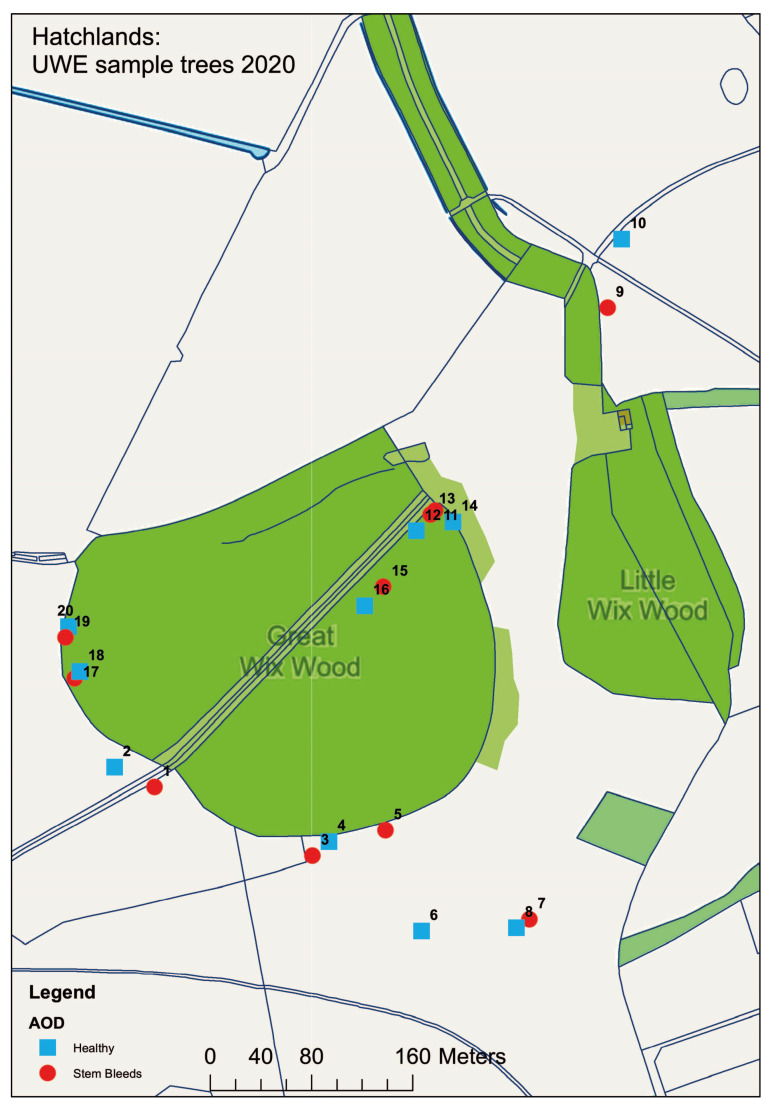
Map detailing the location of samples taken from Hatchlands Park site. Healthy (blue) and AOD-symptomatic (red) oak were selected in a paired model, with odds and evens being paired from 1 and 2, through to 19 and 20. Map made in ArcGIS.

To test if the enrichment method could be used in the isolation of the AOD bacteria from oak field samples, and if these bacterial species are specifically associated with leaves, acorns or the rhizosphere, samples were collected from Hatchlands Park, Guildford, UK. To assess if their association was due to the occurrence of AOD at the site, 60 leaves, 60 acorns and 80 rhizosphere samples were collected from 20 different oak trees, half of which were symptomatic for AOD and the other half were non-symptomatic/healthy. To ensure that environmental and spatial effects were minimised, samples were taken from both the parkland (samples 1–10) and woodland (samples 11–20) sections found at the site. A map detailing where samples were collected can be seen in Figure 2. To ensure that the diseased trees were AOD-symptomatic and that AOD was present at the site, swabs were taken from bleeds where possible and the presence of the AOD-associated bacteria was confirmed using HRM analysis.

### 2.6. HRM Identification of AOD-Associated Bacteria

DNA was extracted from single colonies and spread plates of bacteria from swab samples using alkalic lysis [25]. To confirm the identity of strains, both before and during the experiment, HRM analysis for the species-specific identification of the AOD-associated bacteria was performed using the protocol parameters and primers listed in Table 1, per Bueno-Gonzalez [22]. Primers for the multiplex analysis were added to a mixture with 336 µL of molecular-grade water and 28 µL of each primer to give a working volume of 560 µL, with each primer at a 5 µM working concentration. For each reaction, 7.5 µL of SensiFAST HRM kit Master Mix (Meridian Bioscience, Cincinnati, OH, USA), 2 µL of the primer mix and 4.5 µL of molecular-grade water were added to 1 µL of DNA, giving a final reaction volume of 15 µL. Samples were placed in a Rotorgene real-time PCR machine (Qiagen, Manchester, UK) held at 95 °C for 5 min to denature samples, followed by 30 cycles of 95 °C for 5 sec to denature, 75 °C for 7 sec to anneal primers, a ramp temperature of 73–93 °C with increments of 0.5 °C for a total run time of 37 min, generating species-specific melt curves for the identification of AOD lesion bacteria. Type strains of the four AOD-associated bacterial species were included in each HRM run to generate reference profiles.

### 2.7. Confirmation of Presence of Oak Roots in Rhizosphere Samples

Loop-mediated isothermal amplification (LAMP) was performed on DNA extracted from fine roots from rhizosphere samples to confirm they belonged to oak. Rapid DNA extraction was carried out using the Extract ‘n Amp™ Plant PCR Kit (Sigma-Aldrich) and LAMP performed on the resulting DNA (Bridget Crampton, pers. Comm., Forest Research, UK). Briefly, each 25 µL LAMP reaction contained 1X WarmStart^®^ Colorimetric LAMP Master Mix (New England Biolabs, Hitchin, UK), 1X LAMP primer stock (1.6 µM FIP, 1.6 µM BIP, 0.2 µM F3 and 0.2 µM B3) (Supplementary Table S1) and 1 µL of extracted subterranean root DNA diluted two-fold. Samples were incubated at 65 °C for 60 min in a PCR machine (TECHNE) and reactions were subsequently assessed for colour change. LAMP reactions were positive when a colour change from pink to yellow was seen, while negative reactions had no colour change due to a lack of amplification and remained pink.

## 3. Results

### 3.1. Survival and Isolation of AOD Bacteria from Oak-Related Niches

The use of EE broth was found to be suitable for the re-isolation of all key AOD lesion bacteria from both acorns and leaves following inoculation; these CFU values can be seen in Supplementary Figure S1. *B. goodwinii* and *L. britannica* were initially not re-isolated from the soil after the 0 h time point. However, including a pre-enrichment step in BPW allowed for the recovery of *B. goodwinii* and *L. britannica* over the full six weeks from soil. The four AOD-associated bacteria were successfully recovered from all microcosms each week up to week 3, after which *G. quercinecans* and *L. britannica* were not recovered from acorns in weeks 4 and 5, respectively; *B. goodwinii* and *R. victoriana* were not recovered from acorns in week 6; and *R. victoriana* was not recovered from leaves in weeks 5 and 6. It appears that *B. goodwinii* and *R. victoriana* were no longer viable in acorn niches after week 5, while *R. victoriana* was also not viable in the leaf niche following week 4. The lack of re-isolation of *G. quercinecans* and *L. britannica* from acorns in weeks 4 and 5, respectively, indicates a different issue, as these two species were re-isolated from the same niche in the following weeks.

**Figure 3:**
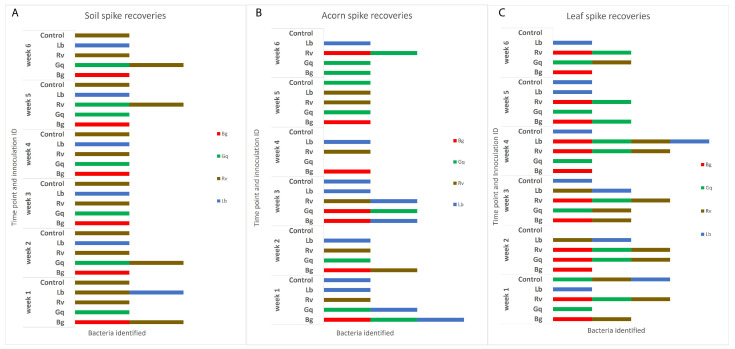
The identities of AOD-associated bacteria recovered from the three inoculated niches, (**A**) indicates the identifications made in soil, (**B**) acorns and (**C**) leaves. The inoculum of each species was adjusted to an OD_600_ of 0.5, which was added to each substrate sample except the controls, to which 1 mL of sterile ¼ strength Ringers was added. Isolation from each sample type took place at weekly intervals over the course of six weeks before identification by HRM in triplicate. Re-isolations are separated by week and the initial inoculated bacteria. Identifications that do not match the original species that were inoculated into spiked substrates can be seen in a number of the columns and the control samples, indicating that AOD bacteria were already present in the majority of samples.

**Figure 4:**
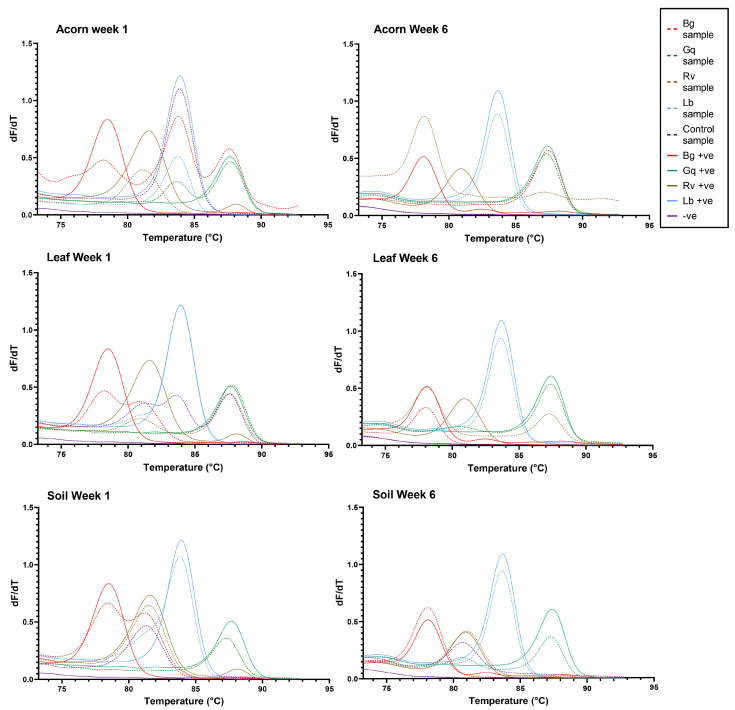
The HRM curves generated for each microcosm at week 1 and week 6 of the experiment. From left to right, the coloured peaks indicate the presence of *B. goodwinii* (Bg), *R. victoriana* (Rv), *L. britannica* (Lb) and *G. quercinecans* (Gq). Solid colour lines represent positive controls, while dashed lines represent samples.

It was also observed that AOD-associated bacteria could be isolated from niches to which they had not been inoculated. *R. victoriana* was isolated from nine leaves and two acorn samples that it was not spiked into, as well as being consistently isolated from the negative control soil samples. In addition to the inoculated microcosms, *G. quercinecans* was isolated from one soil sample, four acorn samples and nine leaf samples, whilst *L. britannica* was isolated from six additional acorn samples and four leaf samples, and *B. goodwinii* was identified in four leaf samples and one acorn. Figure 3 details the AOD-associated bacteria that were re-isolated from soil, acorn and leaves, respectively, per week. The HRM curves for the initial time point and final time point can be seen in Figure 4.

### 3.2. AOD Confirmation of Symptomatic Oak

All AOD-symptomatic oak trees selected for the study had visible stem bleeds, which were recorded during sampling. In cases where the stem bleeds were low enough to reach (all symptomatic trees excluding tree 19), swabs were taken to confirm the presence of AOD-associated bacteria. HRM analysis was performed on nine swabs following processing. *B. goodwinii* was found in five out of seven viable swab samples (two samples were removed due to no growth on agar), while *G. quercinecans* was detected in two (Table 2). However, neither *R. victoriana* nor *L. britannica* were identified from any of the swab samples. This indicated that at least two of the AOD-associated bacteria were present in the stem bleeds, as well as confirming that AOD was present at the site.

### 3.3. LAMP Confirmation of Oak Roots

LAMP analysis of extracted root DNA was used to confirm that the rhizosphere soil adhered to fine roots belonged to oak and not to other plants from the surrounding environment. Each sample was tested in batches by biological sample (four replicates from each tree), and the resulting colour change from pink to yellow was recorded as positive, or negative if no colour change occurred. Supplementary Table S2 shows the result for each sample divided by the sampling location and the health status of the oak. The results demonstrated that 55 of 80 samples contained oak roots, while 27 samples did not, even after two rounds of LAMP. All trees had two or more rhizosphere samples containing oak roots, allowing for two positive samples per tree (total of 40 samples) to be taken forward for the enrichment method and HRM detection of the AOD bacteria.

### 3.4. HRM Analysis of Bacteria from Hatchlands Park Samples

**Figure 5:**
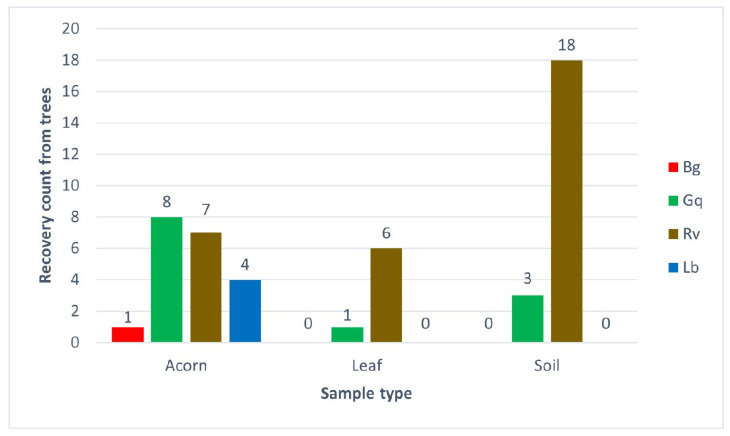
The number of AOD bacterial species recovered from different sample types taken from 20 oak trees at Hatchlands Park, Guildford, UK. The recovery count is the number of trees each bacterium was identified from per sample type; each single identification was confirmed by high-resolution melt analysis in triplicate.

Following the isolation of bacteria with the EE broth method from leaves, acorns and soil samples from Hatchlands Park, multiplex HRM was performed in triplicate to confirm the identity of the isolates. Figure 5 demonstrates the number of times each species was identified from each sample type, while Supplementary Table S3 lists the specific species identifications from each sample.

The results from the Hatchlands Park samples demonstrated similar patterns to the results from the spiking experiment, although the recovery numbers were lower in planta samples. *R. victoriana* was the most environmentally widespread of the AOD-associated bacteria recovered from each niche tested. It was most significantly recovered from soil, with 90% (18 out of 20) of trees tested showing *R. victoriana* as present in their rhizosphere samples. *R. victoriana* was recovered from 35% of acorns and 30% of leaves from the 20 trees sampled; 20% of the identifications from acorns originated from healthy oaks, while 71.4% of the identifications from leaves originated from diseased trees. *G. quercinecans* was equally widely distributed, with identifications made for each sample type. The most frequent recovery of *G. quercinecans* was from acorns, with four identifications from healthy and diseased trees. Only one leaf from a diseased tree and two diseased and one healthy rhizosphere samples returned a positive result for *G. quercinecans*. Both *B. goodwinii* and *L. britannica* were only identified from acorns, with low recovery rates from one healthy tree for *B. goodwinii* and two diseased and healthy trees for *L. britannica*.

## 4. Discussion

Here we provide conclusive evidence that *B. goodwinii*, alongside *G. quercinecans*, *L. britannica* and *R. victoriana* are all capable of survival in soil following inoculation and remain recoverable for six weeks in in vitro experiments. In an earlier study, it was observed that *B. goodwinii* could not be recovered from soil following inoculation, suggesting it enters a viable but non-culturable state and, as such, demonstrates a limited range of environments in which the bacteria can reside in a dormant state outside of its host [23]. Based on these previous results, a recovery method for damaged cells was deemed to be a reasonable approach. Due to the need to test treated food products for pathogens, some enrichment methods that can revive damaged cells already exist, such as *Enterobacteriaceae* enrichment broth [26], which was recently used in the isolation of novel members of the Enterobacterales from soil samples [27]. However, *B. goodwinii* and *L. britannica* were only recoverable in EE broth following the 0 h time point if suspended in BPW first. Whether this is due to the low nutrient environment of soil, leading them to enter the viable but non-culturable state as suggested by Pettifor et al. [23], or due to nutrient shock from the harsh composition of EE broth [28], or their weakened state leaving them to be outcompeted in broth [29], speculation can only be given. Nutrient shock, followed by out competition, seems most plausible due to the ability of prior BPW suspensions to resolve the issue. Aside from this issue, the method appeared to be sufficient for the recovery of all four AOD-associated bacteria from the three niches. The four bacterial species also survived in leaves for the full six weeks (excluding *R. victoriana,* which was not detected after week 4), and in acorns for five weeks (*R. victoriana* and *B. goodwinii* were not recovered following week 5). It is difficult to provide a definitive reason as to why these bacteria were not identified in leaves and acorns at certain time points. One possibility is that, due to the thick waxy exterior of leaves and the solid inner structure of the acorns’ testa, the inoculum did not remain in the plant tissue once injected. However, this seems unlikely considering the successful inoculation of the other two bacterial species. Instead, it is hypothesised that those specific inoculations were outcompeted, either during incubation of the sample or during a stage of the enrichment process during recovery, potentially by other members of the AOD lesion complex. It has been previously demonstrated that the AOD-associated bacteria are capable of co-ordinated behaviour in competitive interactions in vitro [30]. This may explain the lower number of identifications in the field samples and the favouring of certain species based on their competitive fitness, leading to their subsequent detection.

Using the enrichment method on substrate samples collected from field symptomatic and healthy oak, both *R. victoriana* and *G. quercinecans* were identified in oak rhizosphere soil, although *G. quercinecans* was only identified in three of the 20 samples screened compared with the presence of *R. victoriana* in 18 of 20 samples, which demonstrated its widespread distribution. Leaf endophytes showed less of a pattern, with *R. victoriana* being isolated from six of 20 samples and *G. quercinecans* only isolated from one. This is not surprising, as the phyllosphere is seen as a hostile environment for microbial life, with a thick physical barrier to internal entry and inhospitable conditions due to environmental fluctuations [31]. Our study focused on the internal leaf colonisers as surface sterilisation was used, but perhaps further identifications of AOD-associated bacteria would be made if the external leaf colonisers were also considered.

Acorns proved to be a consistent reservoir of the AOD-associated bacteria, with each member being identified at least once. *R. victoriana* and *G. quercinecans* were consistently isolated, while *L. britannica* was isolated four times, twice from acorns from both healthy and diseased oaks. *B. goodwinii* was only clearly isolated once from the acorn of a healthy oak, although given the results from the microcosm experiment, this could be a limitation of the recovery method, rather than an indication of its full distribution. Further attempts with more selective agars for the *Pectobacteriaceae*, such as crystal violet agar [32], might help increase the recovery rate of these bacteria.

While the study was limited to 20 different trees from one site, the consistency of the results has begun to illuminate the life strategies and habitats of the AOD-associated bacteria. While all four species appear to have the ability to survive in a range of environmental niches, *B. goodwinii* and *L. britannica* lack the competitive fitness to survive in the competitive, nutrient-deficient environment that soil offers [29]. *G. quercinecans* and *R. victoriana* are competitive, flexible microbes that appear to have a wide environmental tolerance. This was expected, as *Rahnella* species are well-recorded as being hardy, ecologically diverse bacteria found in numerous environments [33]. Given that *R. victoriana* is isolated from the majority of AOD lesion microbiomes and it is seen to have a secondary role in the disease [10], the findings in the present study support the hypothesis that *R. victoriana* is playing an opportunistic role in symptom development due to its wide dispersal. *G. quercinecans* showed similar dispersal with identifications made from soil, leaves and acorns, but it was less frequently recovered. More importantly, it was significantly more associated with acorns than any other niche, potentially indicating that *G. quercinecans* is an oak endophyte, which would explain the high proportion of lesions samples it is recovered from and the suggested primary role it plays in lesion development [10]. While *L. britannica* and *B. goodwinii* were only identified in a few samples, their sole recovery from acorns strongly implies they are inherited members of the seed endophytic community. Though if true, this does not explain the infrequent isolation of *L. britannica* in lesion samples, except through its poor ability to grow from mixed cultures compared to the other members of the AOD lesion microbiome [34]. However, while all of the AOD-associated bacteria appear to have the ability to be endosymbionts of acorns, the low number of bacteria recovered, specifically of *B. goodwinii*, limits the ability of this study to make conclusive statements as to their origin and method of dispersal.

Nonetheless, the implication of three members of the lesion microbiome being endophytic in acorns, suggesting they are maternally inherited, is concerning. For an endophytic bacterial population to shift to a pathogenic lifestyle, when they are traditionally viewed as plant growth promoters [35], the plant host would have to be severely damaged to the point where homeostasis is disrupted. These concerns were raised when *Brenneria salicis*, the causal agent of watermark disease of willow, was found to reside in symptomless willow trees [36]. It was noted that the ability to stop the spread of *B. salicis* in the same way as a free-living pathogen was no longer possible, and monitoring for its presence no longer enabled early prediction of the disease [37]. Early detection of affected plant hosts and identification systems play crucial roles in understanding ecology and pathogenesis, with the eventual aims of providing treatment prior to symptom development and preventing the spread of disease [38]. Both of these aims become less achievable when the causal agent has an endophytic lifecycle.

While detection and prevention of disease caused by endophytic pathogens is complicated, a new important area of research has arisen to understand how the shift from endophyte to pathogen occurs. For example, *Herbaspirillium rubrisubalbicans* can be both a plant growth promoter and a pathogen of sugarcane. However the mechanisms underlying these dual roles are still poorly understood, and the interactions vary depending on strain genotypes, cultivars and other variables such as the environment [39]. In the case of *B. salicis*, pathogenesis has been attributed to quorum sensing with the production of two *N*-acyl-homoserine lactones correlating with population blooms in diseased wood as well as in vitro studies [36]. The shift from endophyte to pathogen is better understood in some fungal species; for example, high light in the environment triggers the production of H_2_O_2_, causing *Diplodia mutila* to shift to a pathogenic lifestyle on tropical palm [40]. However, as far as the literature shows, little is known about the link between environmental changes affecting trees and the shift of endophytic bacteria to pathogens, be it through quorum sensing or other mechanisms.

Due to the concerning limitations of treating endophytic pathogens, further investigation, coupled with refinements to the enrichment method to increase the recovery of *B. goodwinii*, is required to further reveal the relationship of the AOD-associated bacteria to seed stock in oak. If they were to be inherited endosymbionts from acorns, their role in AOD would be purely a consequence of the environmental predisposition that leads to the tree no longer being able to keep their pathogenic ability at bay. They may be, as many endophytes are, playing the role of latent pathogens that cause disease because the conditions allow for it [41]. This could lead to a stronger understanding of the main causes of AOD and where to focus preventative measures, such as on the aforementioned predisposing and inciting features associated with decline [7].

## 5. Conclusions

The overall findings of this work indicate that the use of selective enrichment for the recovery of members of the Enterobacterales provides a suitable method for assessing the ability of relevant bacteria to survive in different environments. *B. goodwinii*, *G. quercinecans*, *R. victoriana* and *L. britannica*, the four key bacterial species associated with AOD, were shown to survive following inoculation in a number of different environmental niches. *G. quercinecans* and *R. victoriana* appeared to be widespread members of the natural environment, recoverable in greater numbers from all sampled niches, whereas *B. goodwinii* and *L. britannica* were only recovered from acorns in low numbers, suggesting a potential endophytic lifestyle in oak. The possibility that the biotic factors involved in the symptom development of AOD may include both ubiquitous environmental bacterial species and endophytes introduces several complications in the potential management of the disease. Possible treatment via preventative measures becomes limited, and instead efforts would have to focus on controlling the abiotic factors that cause decline to take hold of oak. However, increased understanding of how these bacteria are inherited through seed could lead to the identification of seed stock lineages in which the AOD pathogens are not present, allowing for selective breeding programmes that reduce the occurrence of AOD.

## Figures and Tables

**Table 1:** Primer sequences used in high-resolution melt analysis for AOD bacteria (Bueno-Gonzales, 2022).

Primer	Sequence (5′->3′)
Bgi2F	CGTCAAACTATTTGCTTCCACCCATC
Bgi2R	CGGTATGGGTCGGGACATTTG
Bgi3F	CATCGCGTCCAGCGTCTG
Bgi3R	GCCTATTGCGTGAACGAACTGGATAG
Gqi3F	GCATACGCCTGGTACAGCGC
Gqi3R	CCTTGGCGGGACAGTCTTGC
Rvii1F	GCATCTCGCAGATCGCTGAAAC
Rvii1R	TGGAAGCGGCGGCTGAC
Lbi2F	GGAATCGCTTTACCGTCGCTATTG
Lbi2R	CAAGGTGGTGATGGTGGTCGATC

**Table 2:** High-resolution melt results for the Hatchlands Park lesion swabs.

Tree	Health Status	*B. goodwinii*	*G. quercinecans*	*R. victoriana*	*L. britannica*
1	AOD active bleeds	+	+	-	-
3	AOD active bleeds	+	+	-	-
5	AOD active bleeds	N/A	N/A	N/A	N/A
7	AOD active bleeds	+	-	-	-
9	AOD active bleeds	N/A	N/A	N/A	N/A
11	AOD active bleeds	+	-	-	-
13 *	AOD dry bleeds	-	-	-	-
15 *	AOD dry bleeds	-	-	-	-
17	AOD dry bleeds	+	-	-	-
Totals		5/9	2/9	0/9	0/9

+ = presence of species in the sample; - = no detection; N/A = no growth from samples on agar; * = dry bleeds that were not actively weeping.

## Data Availability

Data are contained within the article.

## Supplementary Materials

The following supporting information can be downloaded at: https://www.mdpi.com/article/10.3390/microorganisms11112789/s1, Figure S1: The colony-forming units from Miles and Misra plates; Table S1: The gene amplified in the LAMP for the identification of oak material; Table S2: Loop-mediated isothermal amplification (LAMP) results; Table S3: The AOD bacteria identified in environmental samples from Hatchlands Park.
