# Supplementary material for: Bacteria Associated with Acute Oak Decline: Where Did They Come From? We Know Where They Go
Source: Microorganisms. 2023 Nov 17;11(11):2789. doi: 10.3390/microorganisms11112789 (PMC10673434; doi:10.3390/microorganisms11112789)
Supplement: Supplementary file 1 [file microorganisms-11-02789-s001.zip › microorganisms-2684624-supplementary.pdf]

# Supplementary Materials

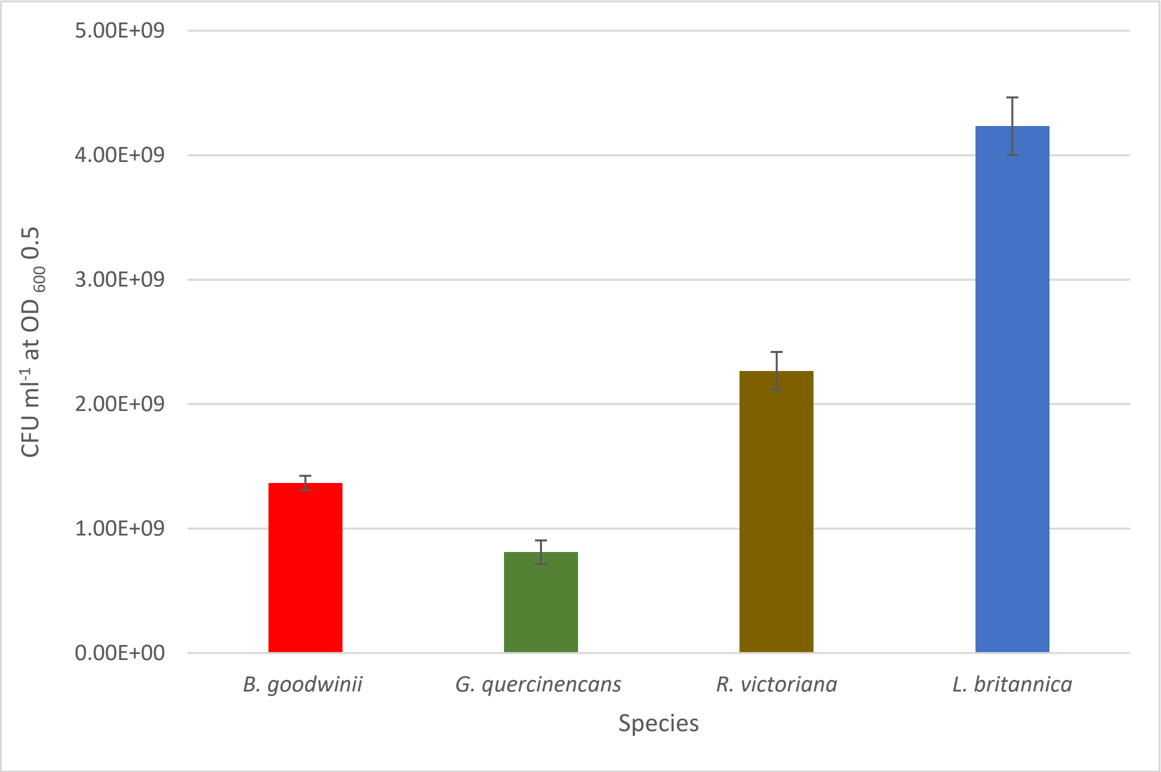


**Figure S1**. The colony forming units calculated from Miles & Misra plates (*n*=3) for each species used in the spiking experiments.

**Table S1**. The gene amplified in the LAMP for the identification of oak material, the primers used to disrupt and amplify that gene and the sequence of the primers used.

| **Gene** | **Primer name** | **Sequence (5'->3')** |
|---|---|---|
| Actin | Actin F3 | AGTTCTTTAAGGACGCCAC |
| | Actin B3 | CCTTGAGGTACTTGCCATG |
| | Actin FIP | TACTTTTTCTTCGTCGTCTTCAGCAGCTGTAAGACTCAGGAGT |
| | Actin BIP | CTCAAACAGAGCGTTTTGGACCGCAGCTTTTCAAGCGGATA |

**Table S2**. Loop-mediated isothermal amplification (LAMP) results. Each cardinal point of each root sample is separated by both its original sample location (parkland or woodland) and its health status (AOD symptomatic or healthy). Samples are positive for oak if a colour change was seen in the first amplification and negative for oak if no change in colour was recorded when undergoing the amplification.

| Hatchlands Park Rhizosphere samples | | | |
|---|---|---|---|
| Parkland | | Woodland | |
| Tree ID | Roots originating from oak | Tree ID | Roots originating from oak |
| H1 | 2/4 | H11 | 2/4 |
| H2 | 2/4 | H12 | 4/4 |
| H3 | 2/4 | H13 | 3/4 |
| H4 | 3/4 | H14 | 3/4 |
| H5 | 3/4 | H15 | 4/4 |
| H6 | 2/4 | H16 | 4/4 |
| H7 | 3/4 | H17 | 3/4 |
| H8 | 2/4 | H18 | 2/4 |
| H9 | 3/4 | H19 | 2/4 |
| H10 | 2/4 | H20 | 3/4 |

**Table S3**. The AOD bacteria identified in environmental samples from Hatchlands Park, Guildford, UK. Bg = *Brenneria goodwinii*, Gq = *Gibbsiella quercinecans*, Lb = *Lonsdalea britannica* and Rv = *Rahnella victoriana*. Samples that could not be collected are marked by N/A and samples without no AOD bacteria identified are marked by -.

| **Tree number** | **Soil** | **Leaves** | **Acorn** |
|---|---|---|---|
| 1 | Rv | N/A | N/A |
| 2 | Rv | - | Gq |
| 3 | Rv | - | Lb, Gq |
| 4 | Rv | - | - |
| 5 | Rv | - | - |
| 6 | Rv | - | - |
| 7 | Rv | Rv | Rv |
| 8 | Rv | Rv | Rv |
| 9 | Rv, Gq | Rv | Rv |
| 10 | Rv | - | Rv |
| 11 | - | Gq, Rv | Gq, Rv |
| 12 | Rv | - | N/A |
| 13 | Rv | - | Gq |
| 14 | Rv, Gq | - | Rv Gq |
| 15 | Rv | N/A | Lb, Gq |
| 16 | - | - | Gq |
| 17 | Rv, Gq | - | N/A |
| 18 | Rv | - | Lb, Rv |

| | | | |
|---|---|---|---|
| 19 | Rv | Rv | N/A |
| 20 | Rv | Rv | Bg, Lb, Gq |
